# 

**Published:** 1898-12

**Authors:** 


					CONTENTS.
Progrei
*s and Practice.
By RonERT
iY " ???? Jrruui?ict3? r>y i\uiskkt
Roxburgh, M.B. Ed., F.R.C.S. Ed.
289
^00ms) Past and Present.
? ~o7 "vuuidj raub miiu riuauuu
v^rir William MacCormac, Bart.,
K.C.V.O.
SOI
"^8?;?a?lcal Cure of Reducible
fi?Fnla. by Kochor's Method of
invagination, with Lateral DIs-
giacement of tho Neck, of the
Sac.
"y J. Lynn Thomas, F.R.C.S.
J J ? i-*YNN X HOMAS, i'.K.U.b.
307
Cholecystotomy.
By T.
C*,.? vmoiecystotomy. uy r.
311
?L-ona
1Vlt Method of Copying
si.u6COiea Meth<
|Phygmograms;
By W. Macphun
OEMdt ",P/?,ua? 15y w- IVlACrHUN
eMpLE, M.B., C.M.Glasg. ...
312
I _
Progress of the Medical Sciences:
Medlclnc.
J. Michell Clarke,
M.D.
314
Surgery.
James Swain, M.D.
318
Laryngology and Rhlnology.
Barclay J. Baron, M.B.
322
Otology.
W. H. Harsant
Reviews of Books
328
Notes on Preparations for the Sick
363
Bristol Medico-Chlrurgical Society
36S
The Library of the Bristol Medlco-
Chirurgical Society 369
Local Medical Notes  378
Scraps
381

				

